# MRI identifies disrupted cerebral development in medulloblastoma patients

**DOI:** 10.1093/braincomms/fcaf090

**Published:** 2025-02-23

**Authors:** Asim K Bag, Joseph Holtrop, John O Glass, Samuel S McAfee, Shengjie Wu, Yimei Li, Matthew Scoggins, Silu Zhang, Giles W Robinson, Amar Gajjar, Tara M Brinkman, Heather M Conklin, Wilburn E Reddick

**Affiliations:** Radiology, St. Jude Children’s Research Hospital, Memphis, TN 38105, USA; Radiology, St. Jude Children’s Research Hospital, Memphis, TN 38105, USA; Radiology, St. Jude Children’s Research Hospital, Memphis, TN 38105, USA; Radiology, St. Jude Children’s Research Hospital, Memphis, TN 38105, USA; Biostatistics, St. Jude Children’s Research Hospital, Memphis, TN 38105, USA; Biostatistics, St. Jude Children’s Research Hospital, Memphis, TN 38105, USA; Radiology, St. Jude Children’s Research Hospital, Memphis, TN 38105, USA; Radiology, St. Jude Children’s Research Hospital, Memphis, TN 38105, USA; Oncology, St. Jude Children’s Research Hospital, Memphis, TN 38105, USA; Oncology, St. Jude Children’s Research Hospital, Memphis, TN 38105, USA; Psychology and Biobehavioral Sciences, St. Jude Children’s Research Hospital, Memphis, TN 38105, USA; Psychology and Biobehavioral Sciences, St. Jude Children’s Research Hospital, Memphis, TN 38105, USA; Radiology, St. Jude Children’s Research Hospital, Memphis, TN 38105, USA

**Keywords:** medulloblastoma, brain development, MRI, DTI

## Abstract

Cognitive decline in survivors of medulloblastoma is commonly attributed to radiation- and chemotherapy-induced brain microstructural alterations. Factors preceding this adjuvant therapy, such as disrupted brain development or resection surgery, may affect brain microstructure but have not been thoroughly explored in medulloblastoma. The aim of this study was to assess cortical thickness and microstructural integrity of the cerebrum prior to adjuvant therapy in medulloblastoma patients. Cross-sectional image data were acquired of medulloblastoma patients (*n* = 30) after surgery but before adjuvant therapy and compared with data from healthy controls (*n* = 35) matched for age range (12–22 years). Biomarkers of microstructural integrity include fractional anisotropy, mean diffusivity, axial diffusivity and radial diffusivity. Thickness, surface area and volume were estimated for parcels of neocortex to evaluate potential morphology differences. Participants with medulloblastoma showed increased diffusivity parameters (mean, axial and radial diffusivity) and decreased fractional anisotropy, within nearly all white and grey matter parcels of the cerebrum, compared with healthy controls. Medulloblastoma participants additionally showed decreased cortical thickness in sub-regions of frontal, parietal, temporal and paracentral cortex. Broad cerebral microstructural alterations in medulloblastoma patients following surgery but before initiation of radiation or chemotherapy suggest that cerebellar insult, by tumour development or tumour resection, likely contributes to compromised integrity of cerebral grey and white matter. Locations of cortical thinning suggest that cerebellar insult may impair normal growth in cerebral regions responsible for executive function, language and attention—cognitive domains typically affected in medulloblastoma survivors.

## Teaser

Bag *et al*. report that medulloblastoma patients exhibit increased diffusivity and decreased fractional anisotropy in most cerebrum regions, along with cortical thinning in targeted areas, before undergoing any adjuvant therapy. These extensive microstructural changes suggest that cerebellar damage undermines the integrity of both cerebral grey and white matter.

## Graphical Abstract

**graphical abstract:**
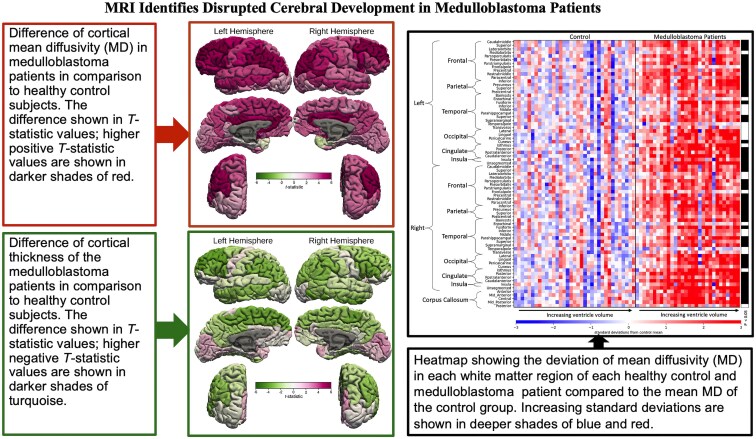

## Introduction

Medulloblastoma (MB) is the most common primary malignant brain tumour of childhood, which arises from the cerebellum.^1^ Cognitive impairment leading to poor quality of life is a common burden for MB survivors and is generally attributed to adverse effects of adjuvant therapy [craniospinal irradiation (CSI) and chemotherapy],^2-4^ which is thought to be mediated by compromised brain integrity, apparent in the form of regional^3-6^ and global^6,7^ white matter damage, altered structural brain connectivity,^8,9^ cortical thinning^10^ and reduction of the volume of the hippocampus.^3,11^ While chemotherapy and CSI are most often the prevailing factors leading to cognitive impairment in patients with MB,^1,2,12^ efforts to understand how their impact is mediated by specific changes in brain integrity are complicated because the process of tumourigenesis, secondary effects of relatively large tumour size and surgery can also potentially alter the normal brain development in these patients due to tumour mass effect, altered cerebello-cerebral circuit development and surgical injuries in posterior fossa. So far, there has not been any study designed to explore the effects of tumour and surgery in normal brain development.

In previous cross-sectional studies using parametric maps and tract-based spatial statistics analysis of diffusion tensor imaging (DTI), we have demonstrated microstructural alterations in frontal lobe white matter, following surgery in MB patients but before CSI or chemotherapy.^5^ As the cerebellum is densely inter-connected with the cerebrum through afferent and efferent pathways, and as both the processes of tumourigenesis and surgical resection of MB can impair the normal cerebello-cerebral pathways, we hypothesized that patients with MB would have diffuse grey matter (GM) and white matter (WM) microstructural alterations throughout the cerebrum, even before CSI and chemotherapy. We tested this hypothesis by examining cortical thickness and DTI measures of microstructural integrity of GM and WM throughout the cerebrum in patients with MB following resection but before start of any other therapy by comparing these radiological parameters with healthy control subjects without any prior cancer history, matched for age range.

## Materials and methods

### Study design and participants

**Figure 1:**
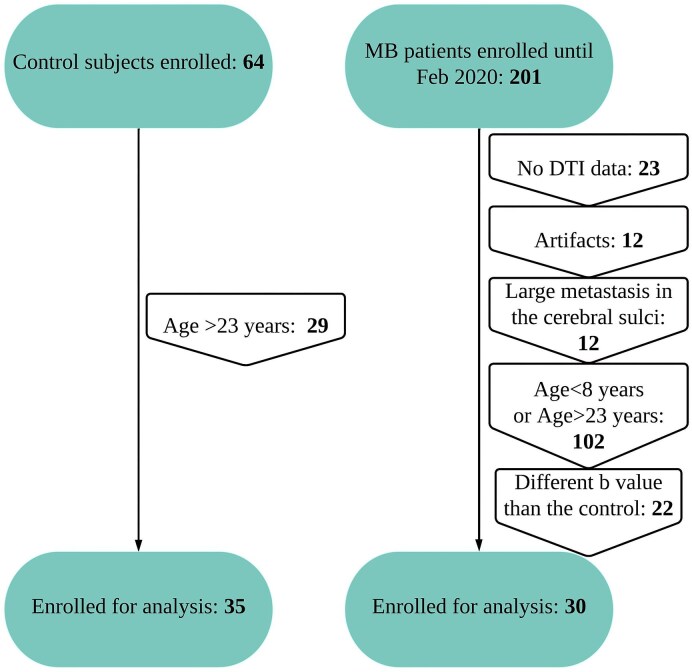
**Participant enrolment flowchart.** Flowchart shows participant enrolment in the study.

Subjects in our control group were included from a study performed at St. Jude Children’s Research Hospital on healthy children and adolescent English-speaking subjects enrolled on the FACES arm of the SJLIFE protocol (NCT00760656) as a community control without any (i) history of childhood cancer or cancer-related therapy, (ii) history of a genetic disorder/neurodevelopmental condition associated with neurocognitive or social impairment, (iii) history of head injury associated with neurocognitive impairment and (iv) diagnosis of a serious psychiatric condition associated with neurocognitive or social morbidities: (*n* = 35; age range, 12–22 years; mean age, 18.0 years, 17 male). The patient group was included from MB patients enrolled in a therapeutic trial investigating clinical and molecular risk–directed therapy for newly diagnosed MB patients (SJMB12 and NCT01878617) until February 2020. Criteria for inclusion of MB patients in this study included the following: (i) patients’ age between 12 and 22 years (limited to correspond more closely to the available healthy control subjects and to eliminate the use of sedation) (Supplementary Fig. 1A), (ii) absence of any artefact on imaging, (iii) imaging obtained before the start of CSI and chemotherapy, (iv) imaging parameters are identical to those of the healthy control subjects and (v) absence of any large (>1 cm) metastasis in the cerebral sulci (Fig. 1). The selection criteria resulted in 30 patients in the MB group (age range, 12–21 years; mean age, 15.8 years, 19 male). The patient demography, tumour location and sub-type and duration of symptoms at presentation are included in Supplementary Table 1. The control subjects and the MB patients were also matched for IQ (Supplementary Fig. 1B and Table 2). Almost all controls and patients were right handed (Supplementary Table 3). Both the studies were approved by the institutional review board at St. Jude Children’s Research Hospital; parents, guardians or patients or healthy subjects provided written informed consent, and patients 7–13 years of age provided assent before study enrolment. The time difference between the date of surgery and the MRI acquisition was also recorded for those with MB.

### Image acquisition

Imaging of all the participants, healthy control and MB patients was performed using the same magnets and identical imaging protocol. Imaging of MB patients was performed after resection of the tumour and prior to start of adjuvant therapy. All imaging was performed on Siemens 3T platform (Prisma or Skyra, Siemens Medical Systems; Supplementary Table 4) and included a 3D sagittal T1 MPRAGE image and DTI. Parameters for the 3D T1 MPRAGE were as follows: repetition time (TR) = 1980 ms; echo time (TE) = 2.26 ms; inversion time (TI) = 1100 ms; flip angle = 15° and acquisition matrix: 256 × 256, 1 mm^3^ isotropic.

DTI was acquired using bipolar diffusion-encoding gradients to reduce gradient-induced eddy currents by using a double-spin echo, simultaneous multi-slice, multi-echo planar imaging pulse sequence^13^: TR = 4000 ms; TE = 78.6 ms, b = 0 and b = 1500 s/mm^2^ at 64 directions; 1.8 mm isotropic resolution; 84 slices; multi-band factor: 4 and acquisition matrix: 128 × 112. The DTI acquisition was performed twice, once with phase encoding in posterior to anterior direction and a second time with the phase encoding in anterior to posterior direction.

### Image analysis

#### Parcellation of the cerebral structures

The T1 image was used to segment the brain for all participants by using FreeSurfer 6.0 (https://surfer.nmr.mgh.harvard.edu/) with segmentations being checked and manually edited, when necessary, at multiple control points within the FreeSurfer processing pipeline. Forty-one regions from each hemisphere and 5 midline regions (a total of 87 regions) from the Desikan–Killany atlas were used for analysis to extract metrics for each segmented region of GM area, GM thickness, GM volume and WM volume.^14^ Additionally, the volume of sub-cortical GM structures was obtained along with diffusion metrics from each segmented region as described below. The volume of the lateral ventricles was also obtained from the FreeSurfer segmentation with the cumulative value of the right and left ventricles used as a surrogate metric for hydrocephalus in the analysis. Each parcelled segment in each subject was manually verified for accuracy. If there was any discrepancy, then the parcellated segment was manually corrected.

#### Processing of diffusion tensor imaging

Processing steps included correction of susceptibility-induced distortions of the echo planar imaging acquisition using the FSL tool topup^15,16^ and correction for head movements and eddy current–induced distortions using the FSL tool eddy.^17^ DTI parameters [fractional anisotropy (FA), mean diffusivity (MD), axial diffusivity (AD) and radial diffusivity (RD)] were estimated by using the FSL tool dtifit.^15-17^

#### Image registration and diffusion tensor imaging parameter extraction

The distortion-corrected mean b = 0 diffusion image and T1 image were registered by using a linear transform between the diffusion and T1 images using FSL.^18-20^ The transformations were visually inspected to ensure accuracy and were adjusted if necessary. Each segmented brain region was transformed into the DTI imaging space using FSL, and the mean DTI parameters were calculated for each segmented region.

#### Assessing resected cerebellar volume

For the patient group, the volumes of the resected portion of the cerebellum were calculated by an automated template normalization and comparison approach.^21^ Resection maps were inspected and adjusted when necessary.

#### Statistical analysis

Altogether, we investigated 12 parameters in regions of the brain: GM volume, GM thickness, GM surface area, WM volume, WM FA, WM MD, WM RD, WM AD, GM FA, GM MD, GM RD and GM AD. To detect group differences, a general linear model (GLM) was used to investigate the difference of each parameter between two groups (Controls and Patients) in each region after controlling for age (the mean age of our control subjects was higher than the study subjects, and age is known to influence DTI parameters and the mean) and ventricular volume (ventricular volume of our study subjects was higher than the control subjects, and ventricular volume is known to influence DTI parameters). The *P*-values for one specific parameter from all regions for group difference between controls and patients were adjusted among regions using the Benjamini–Hochberg procedure for a false discovery rate of 5%. In short, *P*-values were corrected for each individual parameter across all regions.^22^ To identify potential causes, a GLM was used in the MB group to detect a relationship between the 12 investigational parameters and the resected cerebellar volume and the time since surgery after controlling all these parameters for age and volume of the lateral ventricles. The *P*-values for surgery effect were adjusted for multiple comparisons across all regions for each parameter independently.

## Results

### Alteration in microstructural integrity of the different grey matter areas

**Figure 2:**
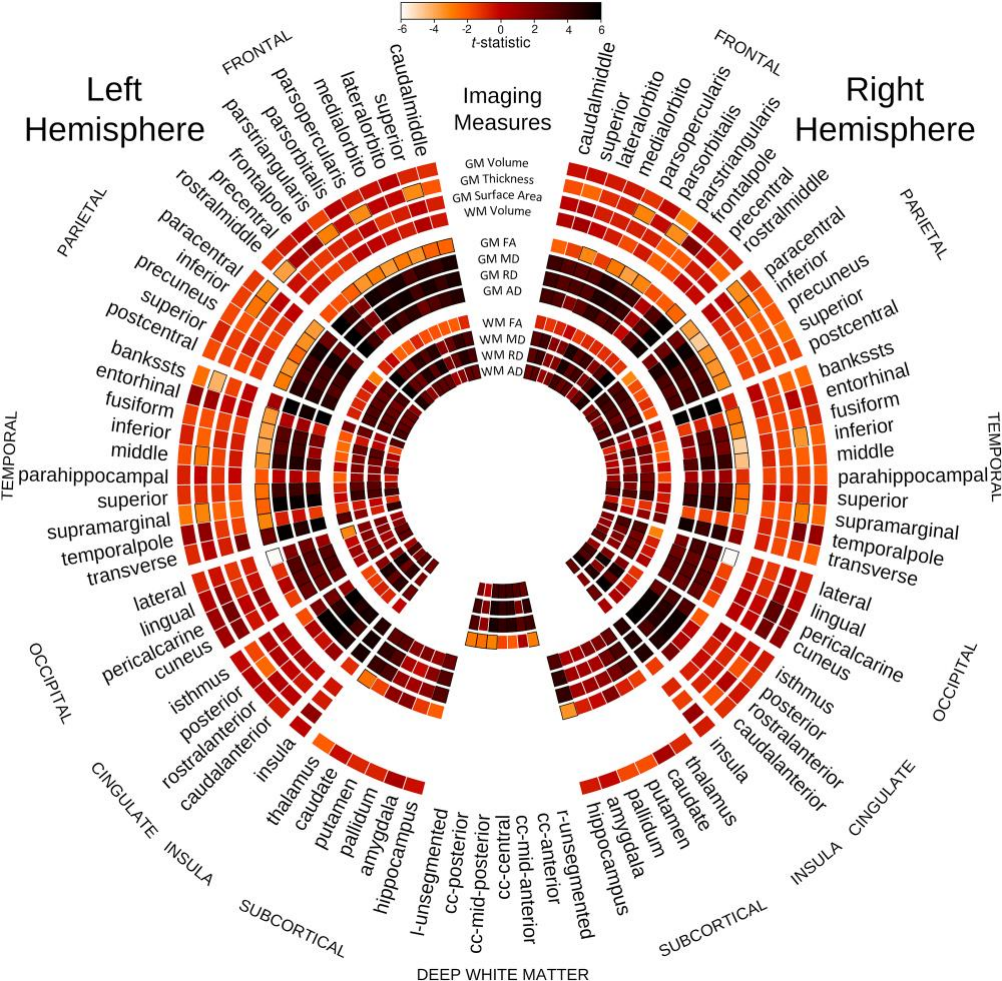
**Group comparison of control subjects and MB patients.** Group comparison of healthy control subjects and MB patients after controlling for age and ventricular volume for the 12 parameters assessed across brain regions. The colour bar demonstrates the mapping of the *t*-statistic from white (Negative 6) to black (Positive 6) through varying shades of orange. Each sector of the circle represents a different region. Each ring of the figure shows *t*-statistic for a different metric, as demonstrated at the 12 O’clock position of the figure. Specific GM and WM regions are outlined in black if the difference between control and MB groups is statistically significant (*P* < 0.05).

**Figure 3:**
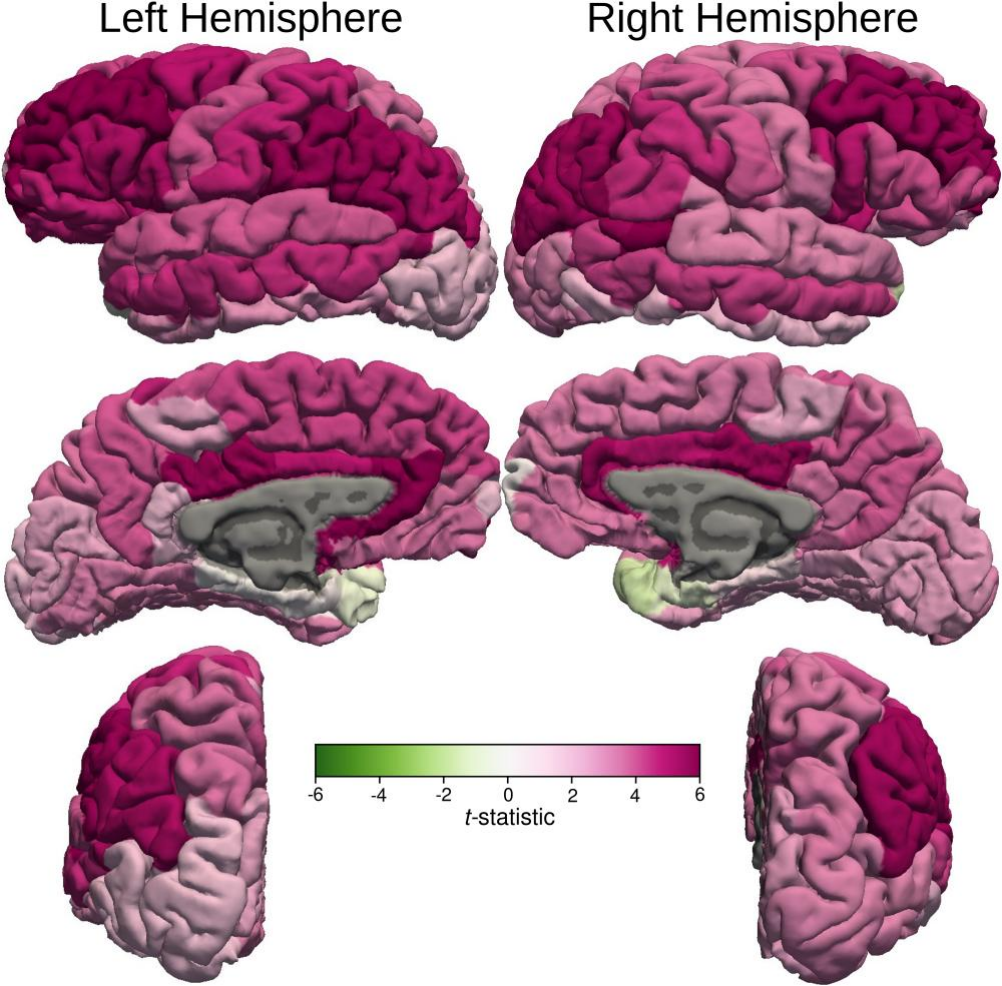
**Cortical MD *t*-statistic displayed on cortical surfaces.**  *t*-statistic values of the cortical MD are displayed on cortical surfaces. The colour bar demonstrates the mapping of the *t*-statistic from green (Negative 6) to magenta (Positive 6) through varying shades. Darker shades of magenta in the middle frontal gyrus, precuneus, posterior aspect of the medial prefrontal cortex and cingulate gyri indicate maximum increase in MD compared with the healthy control subjects. The changes seen in frontal poles and temporal poles, entorhinal and parahippocampal gyri were not statistically significant.

All diffusivity parameters (MD, AD and RD) in almost all the sub-regions of the bilateral frontal, parietal, temporal including bilateral hippocampi and occipital lobe GM in MB patients were significantly higher than those in the healthy control participants after controlling for age and ventricular volume (Fig. 2; Supplementary Figs 2–4). FA in MB patients was lower in many of these areas (Supplementary Fig. 5). The group difference in cortical MD between the MB patients and the healthy controls is visualized on a surface rendering of the brain (Fig. 3).

### Alteration in microstructural integrity of the different white matter areas

All the diffusivity parameters (MD, AD and RD) in almost all the sub-regions of the bilateral frontal, parietal, temporal and occipital lobe WM in MB patients were significantly higher than those in the healthy control participants after controlling for age and ventricular volume (Fig. 1; Supplementary Figs 6–8). FA in MB patients was significantly lower in six of these areas (Fig. 2; Supplementary Fig. 9).

### Alteration in cortical thickness, grey matter volume and surface area

**Figure 4:**
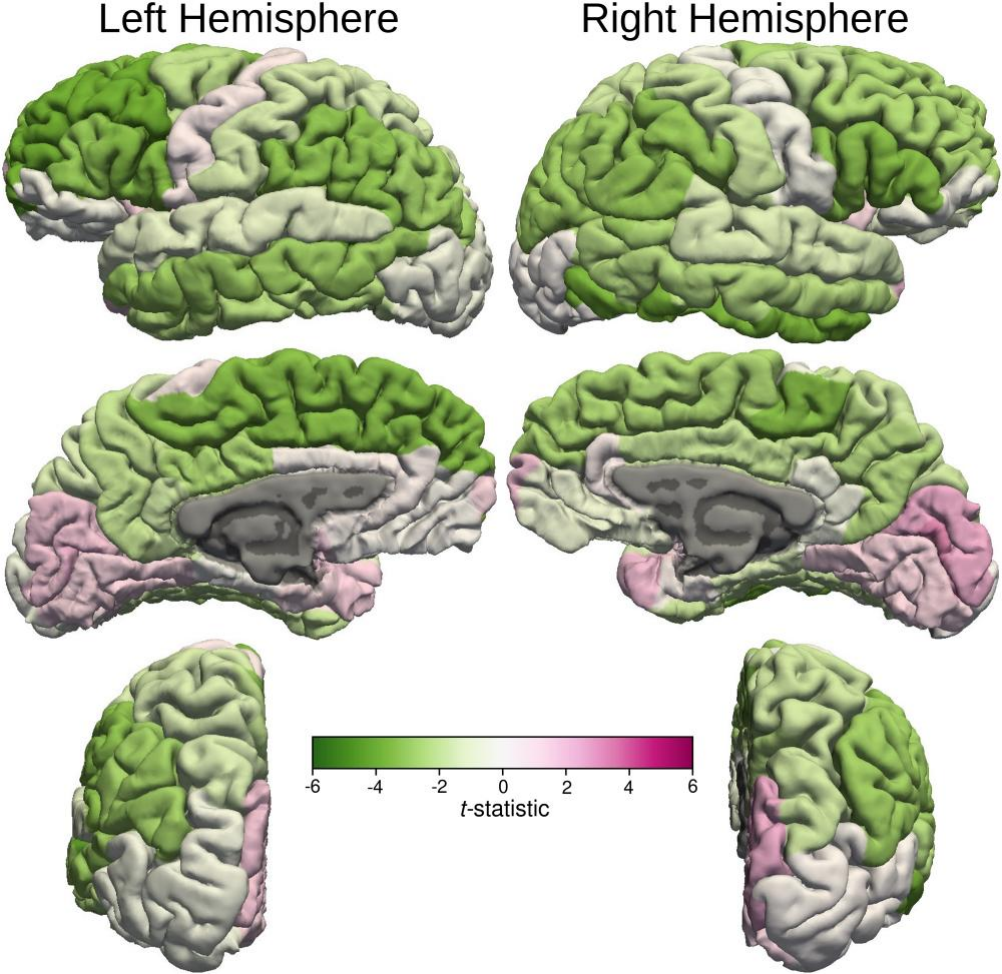
**Cortical thickness *t*-statistic displayed on cortical surfaces.**  *t*-statistic values of the cortical thickness are displayed on cortical surfaces that demonstrate bilateral asymmetry. The colour bar demonstrates the mapping of the *t*-statistic from green (Negative 6) to magenta (Positive 6) through varying shades. Darker shades of green indicate statistically significant decrease in cortical thickness compared with the healthy control subjects in the left superior and inferior frontal, left paracentral lobule, left inferior parietal, left supra-marginal and middle temporal gyrus, right inferior frontal gyri, right paracentral lobule, right inferior parietal, right inferior temporal and right supra-marginal gyri. There was also a statistically significant increase in cortical thickness compared with the healthy control subjects in the right peri-calcarine cortex.

After controlling for age and ventricular volume, the cortical thickness of left superior frontal, left rostral middle frontal cortex, bilateral pars opercularis, pars triangularis, bilateral paracentral lobule, bilateral inferior parietal lobule, left middle and right inferior temporal cortex, cortical areas around left superior temporal sulcus and bilateral supra-marginal gyri of the MB patients were significantly thinner than those of the healthy control subjects (Figs 2 and  4) Volumes of the left and right supra-marginal gyrus and cortical areas around superior temporal sulcus were lower in those with MB than in the healthy control participants, after controlling for age and ventricular volume. Similarly, the surface area of left supra-marginal gyrus and right pars triangularis, right frontal pole and right superior parietal lobe of those with MB were thinner than those of the healthy control participants. Although global WM volume was lower in those with MB than in the healthy control group, the difference was not statistically significant in any WM areas. Also, cortical thickness of all the bilateral occipital lobe areas was more in MB patients compared with control subjects.

### Analysis of association of interval between surgery and MRI acquisition and resected cerebellar volume within MB group

The mean interval (±standard deviation) between the surgery and the date of MRI acquisition was 19.97 ± 7.01 days (Supplementary Fig. 10). Except for transverse temporal gyrus and rostral middle frontal gyrus, the GLM among patients with MB did not demonstrate any association between the time interval (between surgery and MRI acquisition) and pan cerebral microstructural alterations or altered cortical thickness after controlling for age and volume of the lateral ventricles (Supplementary Table 5). The median cerebellar resection volume was 2359 mm^3^ (range: 0–10 491 mm^3^). The most common areas of surgical resection included the peri-ventricular parenchyma (including roof of the fourth ventricle, nodulus, areas of the dentate nuclei and putative beds of the fastigial nuclei) and midline inferior vermal tissues. Resection of the nodulus and midline inferior vermis was noted in 24 of the 30 patients (Supplementary Fig. 11). The GLM did not detect any association between resected cerebellar volume and pan cerebral microstructural alterations or altered cortical thickness after controlling all these parameters for age and volume of the lateral ventricles.

### Brain and ventricular volumes

Due to hydrocephalus, ventricles were significantly larger in patients with MB (Supplementary Figs 12 and 13); however, brain volume of the MB patients and the healthy control participants was not significantly different suggesting comparable brain volumes between the groups (Supplementary Figs 14 and 15).

## Discussion

We have demonstrated extensive microstructural alterations characterized by decreased FA and increased MD, RD and AD in cerebral GM and WM regions in MB patients after surgery but before radiation or chemotherapy, compared with controls after controlling for age and ventricular volume as a surrogate for hydrocephalus. Our observation of reduced FA aligns with prior research that investigated diminished FA values in the frontal lobe WM using tract-based spatial statistics, employing a similar study design.^5^ Additionally, the extensive diffusion changes in both the GM and WM of the cerebrum are similar to those attributed to CSI-induced brain microstructural changes characterized by decreased FA and increased MD, RD and AD in the affected WM.^5,23^ Similar changes have been reported in GM in pre-clinical studies.^24^ The results of our study show that the collective impact of pre-CSI factors on cerebral microstructure is non-trivial and may complicate or confound the interpretation of diffusion-based changes that have been associated with CSI at the end of therapy.^6,7^Cortical thinning was also observed prior to CSI, although differences in cortical thickness were more circumscribed than differences in diffusion metrics. The current study is designed to examine the impact of multiple factors that precede cancer therapies; therefore, this disparity may reflect the differential impact of individual factors on brain microstructural integrity versus the growth and development of cortical GM volume. The near ubiquitous difference in diffusion metrics is indicative of a mechanism that impacts the cerebrum broadly, if not uniformly, which may include increased intracranial pressure or reaction to posterior fossa surgery.

The more circumscribed differences in cortical thickness, along with the assumption that cortical thickness is not likely to change appreciably between surgery and postoperative imaging, suggests a different mechanism. We hypothesize that cortical thinning may be driven by attenuated cerebellar output, leading to insufficient activation-dependent trophic stimulation in thalamo-recipient layers of cerebral cortex mediated by ‘inter-regional morphogenesis’ during embryonic and post-natal development, a process in which development of one region is thought to effect development of the other.^25^ This aligns with findings from studies that observed abnormal cortical development following diffuse injury to functionally associated regions of the premature cerebellum.^26,27^ The distribution of cortical thinning of our study roughly aligns with the known distribution of cerebellar projections to granular layers of cortex, which further supports our hypothesis about the thinning mechanism. Ascending outputs of the cerebellum generally activate higher-order and associative thalamic nuclei (as opposed to sensory relay nuclei) to facilitate communication between the appropriate cortical areas for a given task,^28,29^ and the areas we found to have the greatest negative impact align closely with association areas receiving modulatory thalamic input.^30-32^ Interestingly, thinning in primary motor cortices was conspicuously absent despite its dense input from the cerebellum, which we propose may be due to the lack of a dedicated thalamo-recipient cortical layer in the ‘agranular’ motor cortex.^33^ Similarly, anterior cingulate and orbitofrontal cortices are considered ‘dysgranular’^34^—lacking a well-defined thalamo-recipient layer—and were unchanged in the MB group. Primary visual cortical areas were similarly unaffected or even appeared to have increased thickness. The visual cortex possesses a well-defined granular structure,^33^ but the absence of cortical thinning is consistent with our hypothesis given the lack of known reciprocal connections between the cerebellum and primary visual cortex.^35,36^

Executive functions, working memory, attention and processing speed are key domains that are typically affected in MB survivors.^2^ In current clinical practice, CSI is often conceptualized as the major contributor of all these cognitive impairments; however, similar impairments are also seen in cases where cerebellar outflow tracts are severely injured during posterior fossa surgery in cerebellar cognitive affective syndrome^37^ and in cerebellar mutism syndrome^38-41^ The results of our study suggest that diffuse cerebellar injury caused either by tumourigenesis or resection or combination of both factors caused widespread cerebral microstructural changes, which suggests attenuated cerebellar output may ultimately contribute to persistent altered cognitive functions in MB survivors, although this impact remains difficult to untangle from the impact of unavoidable subsequent CSI.

This study has some inherent weaknesses that should be considered. Both hydrocephalus and increasing age can influence DTI parameters and cortical thickness. In this study, we compared the DTI parameters and the cortical thickness between the MB patients and the healthy control subjects after controlling for age range and ventricular volume as a surrogate of hydrocephalus to mitigate the influence of these factors on DTI parameters. Hydrocephalus, when present in our study subjects, was due to obstruction of the CSF flow by the tumour. We acknowledge that obstructive hydrocephalus significantly alters CSF dynamics, including glymphatic flow, which adds a layer of complexity to interpreting the neuroimaging findings. The DTI model has inherent limitations in fully capturing the complexities of tissue microstructural changes associated with altered water diffusion resulting from disrupted CSF dynamics in obstructive hydrocephalus, and we recognize this as a limitation of our study. Although both 3T Prisma and 3T Skyra systems were used to scan the study and control subjects, we did not see any statistically significant difference between the FA and MD of the entire GM and WM in the control subjects; this is in alignment with previous report of cross-scanner compatibility of DTI parameters.^42^ Importantly, we could not investigate reversibility of these extensive microstructural alterations and cortical thinning that preceded CSI and chemotherapy. Since all MB patients are treated with CSI and chemotherapy following surgery and these factors are known to have negative impacts on cognition, it is not feasible to determine if pre-CSI and pre-chemotherapy microstructural alterations and cortical thinning play a role in the final cognitive outcomes of MB survivors. Although the age range between the MB patients and control subjects was matched, the controls were generally older. This may have influenced the DTI parameters, as these parameters typically change during adolescence due to ongoing WM development, and the authors recognize that this could be a limitation.^43^ To mitigate this potential bias, we considered differences in GM and WM parameters to be significant only if they exceeded 2 SD from the control group’s mean. Similarly, there is evidence of sexual dimorphism in WM development, particularly at younger ages, with differences being more pronounced around 8–9 years and diffusion measures in males and females tending to converge between 10 and 14 years.^44^ In our study, the youngest participants in both the control and study groups were 12 years old, by which point the sexual differences in DTI metrics are reduced, and as a result, sex was not included in the GLM model. However, this could potentially be a limitation. Additionally, due to unavailability of the data, we could not investigate the effects of steroids and anaesthesia on these changes, which can potentially influence the DTI parameters, neither could we establish if the imaging changes are due to surgery or tumour by itself or a combination of the two.

In summary, our study revealed the presence of broad microstructural alterations and cortical thinning in specific brain regions among MB patients before the initiation of radiation or chemotherapy. These findings imply that cerebellar damage, resulting from tumour development or resection or a combination of both, may be linked to altered cortical organization in specific brain regions and broad microstructural alterations, potentially due to disruptions in brain development or other mechanisms. The precise cognitive implications of these changes still require further investigation. Our research provides valuable insights into the early-stage effects of MB on brain structure and opens new avenues for future investigations into cognitive outcomes in MB survivors.

## Supplementary Material

fcaf090_Supplementary_Data

## Data Availability

The authors are committed to making the data and materials related to this study available to readers upon reasonable request to the corresponding author.
