# Supplementary material for: MRI identifies disrupted cerebral development in medulloblastoma patients
Source: Brain Commun. 2025 Feb 23;7(2):fcaf090. doi: 10.1093/braincomms/fcaf090 (PMC11879392; doi:10.1093/braincomms/fcaf090)
Supplement: fcaf090_Supplementary_Data [file fcaf090_supplementary_data.pdf]

Supplementary Materials

MRI Identifies Disrupted Cerebral Development in Medulloblastoma Patients

[1*]Asim K Bag, [1*]Joseph Holtrop, [1]John O Glass, [1]Samuel S. McAfee, [2]Shengjie Wu, [2]Yimei Li, [1]Matthew Scoggins, [1]Silu Zhang, [3]Giles Robinson, [3]Amar Gajjar, [4]Tara Brinkman, [5]Heather Conklin, [1]Wilburn E Reddick

TABLE OF CONTENTS

Supplementary Figure 1. Age and IQ distribution of study participants. 3

Supplementary Figure 2. Heatmap of MD in gray matter regions. 4

Supplementary Figure 3. Heatmap of AD in gray matter regions. 5

Supplementary Figure 4. Heatmap of RD in gray matter regions 6

Supplementary Figure 5. Heatmap of FA in gray matter regions 7

Supplementary Figure 6. Heatmap of MD in white matter regions 8

Supplementary Figure 7. Heatmap of AD in white matter regions 9

Supplementary Figure 8. Heatmap of RD in white matter regions 10

Supplementary Figure 9. Heatmap of FA in white matter regions 11

Supplementary Figure 10. Heatmap showing the areas of the cerebellum resected in the MB patients 12

Supplementary Figure 11. Histogram of the interval between the surgery and the date of MRI acquisition. 13

Supplementary Figure 12. Histogram of ventricular volume in MB subjects 14

Supplementary Figure 13. Scatter plot of ventricular volume versus age 15

Supplementary Figure 14. Brain volume distribution of study participants 16

Supplementary Figure 15. Scatter plot of brain volume versus age 17

Supplementary Table 1. Demography and tumor characteristics 18

Supplementary Table 2. Comparison of the IQ Value between the Control and MB patients 20

Supplementary Table 3. Distribution of the magnets for MRI acquisition 21

Supplementary Table 4. Significant association between time interval (between surgery and MRI acquisition) and specific microstructural alterations in specific brain regions. There is no significant association between time interval and any DTI parameters in rest of the brain areas. (FA, fractional anisotropy; MD, mean diffusivity, RD, radial diffusivity) 21

Supplementary Table 5. Handedness of the control subjects and MB patients 21

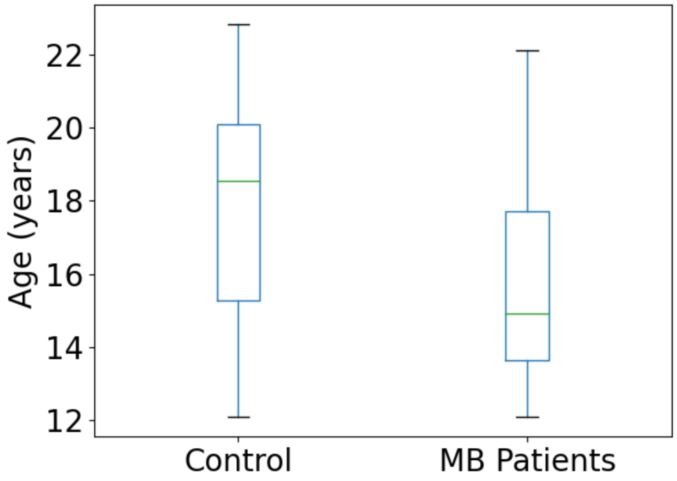

Supplementary Figure 1a. Age distribution of healthy control subjects and medulloblastoma (MB) patients. A t-test indicated the age difference between the groups was statistically significant. ($p<0.05$)

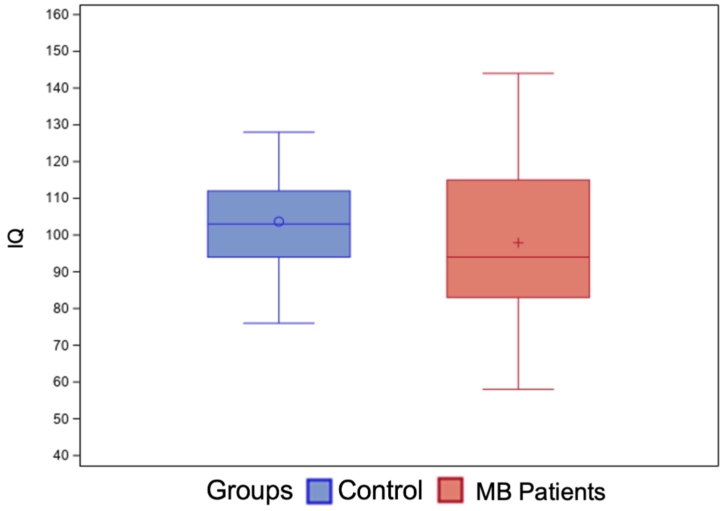

Supplementary Figure 1b. IQ distribution of healthy control subjects and medulloblastoma (MB) patients. A t-test indicated the difference between the groups was not statistically significant. ($p=0.35$)

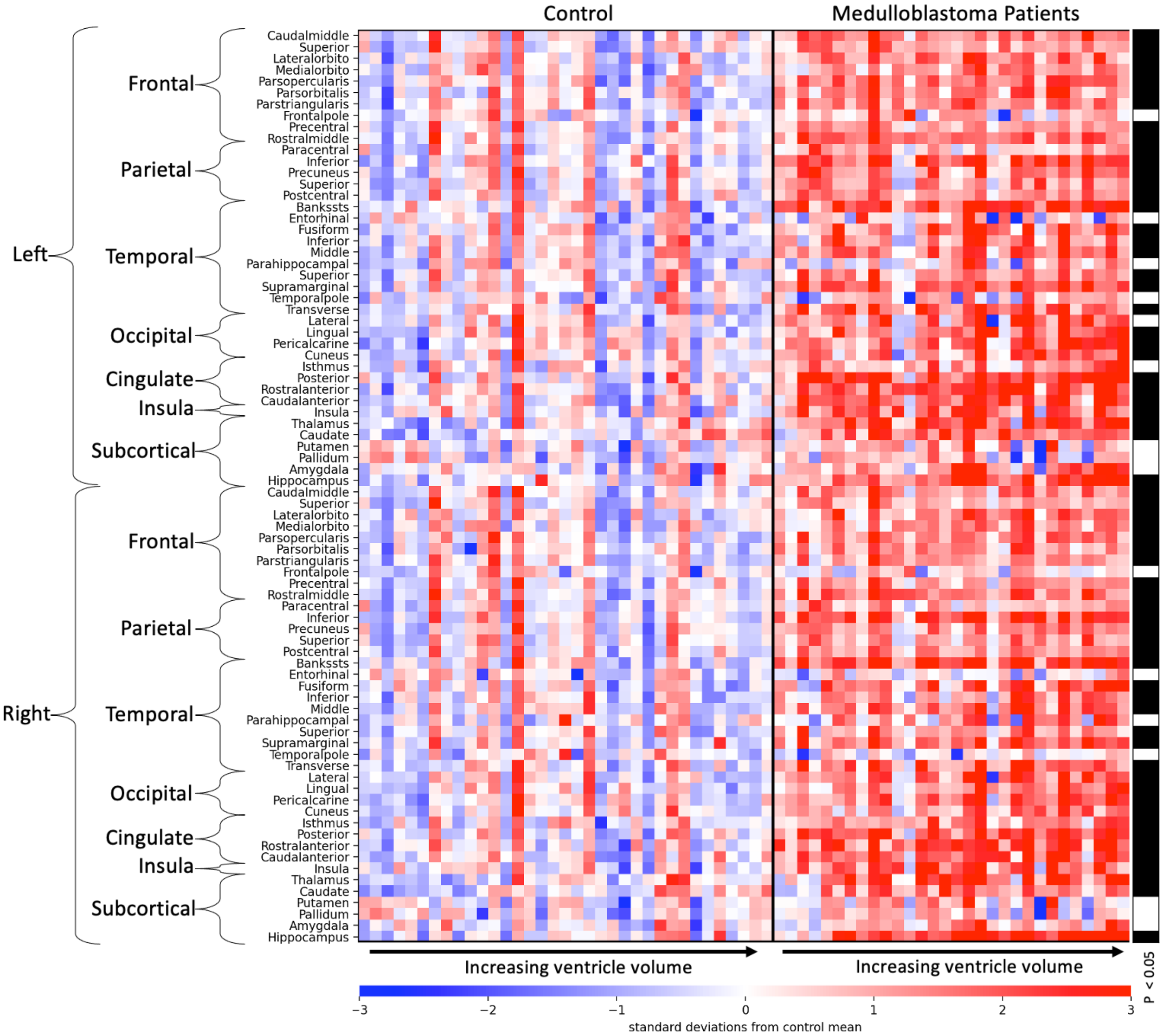

Supplementary Figure 2. Heatmap showing the deviation of mean diffusivity (MD) in each gray matter region of each healthy control and MB patient compared to the mean MD of the control group. In each group, subjects are aligned from left to right from smallest ventricular size to the largest ventricular size. The intensity of blue and red coloring indicates the standard deviation from the mean of the control group. The black color along the right side indicates that the difference from the mean of the control group is statistically significant ($p<0.05$) using a GLM that controls for age and ventricular volume.

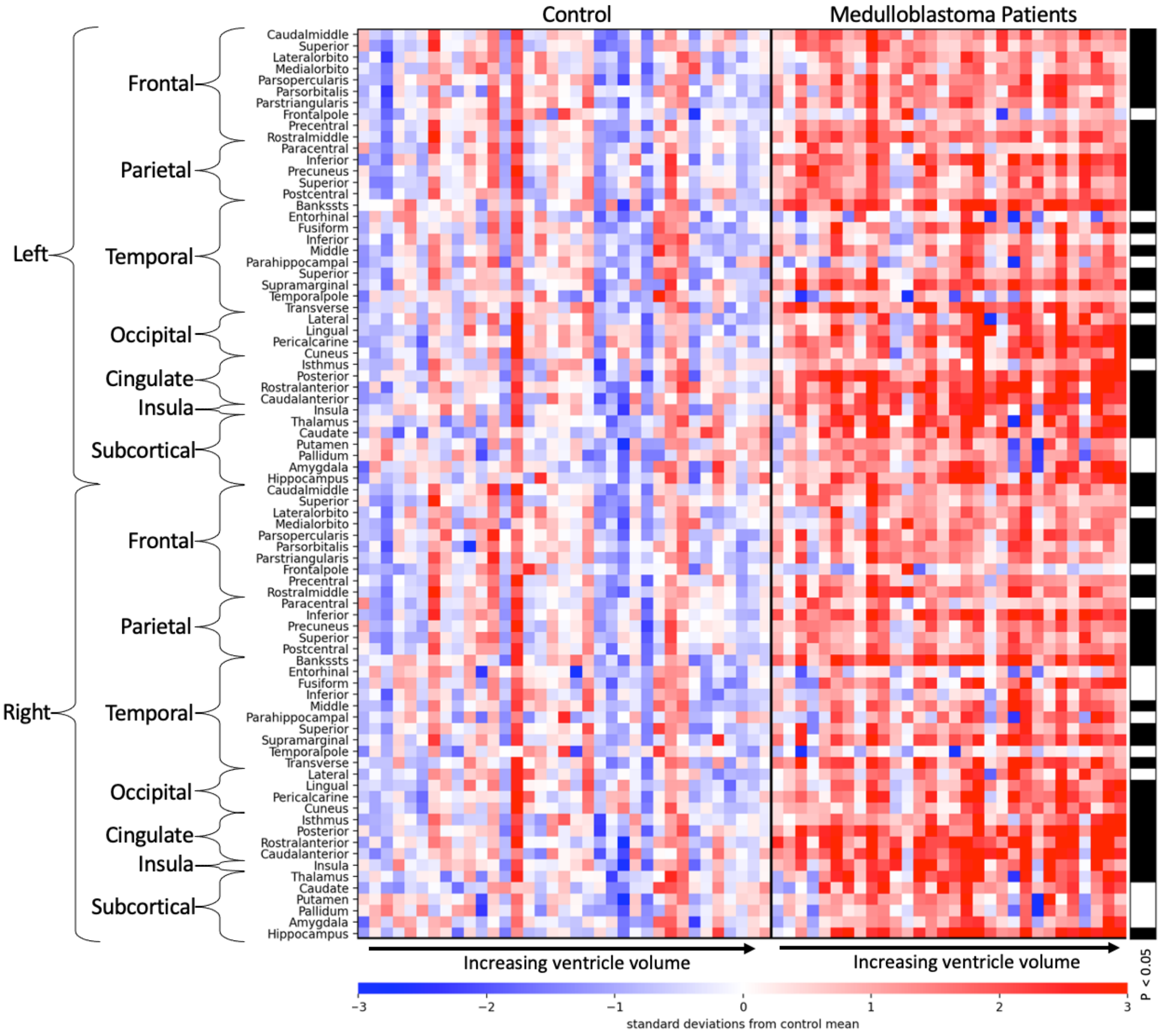

Supplementary Figure 3. Heatmap showing the deviation of axial diffusivity (AD) in each gray matter region of each healthy control and MB patient compared to the mean AD of the control group. In each group, subjects are aligned from left to right from smallest ventricular size to the largest ventricular size. The intensity of blue and red coloring indicates the standard deviation from the mean of the control group. The black color along the right side indicates that the difference from the mean of the control group is statistically significant ($p<0.05$) using a GLM that controls for age and ventricular volume.

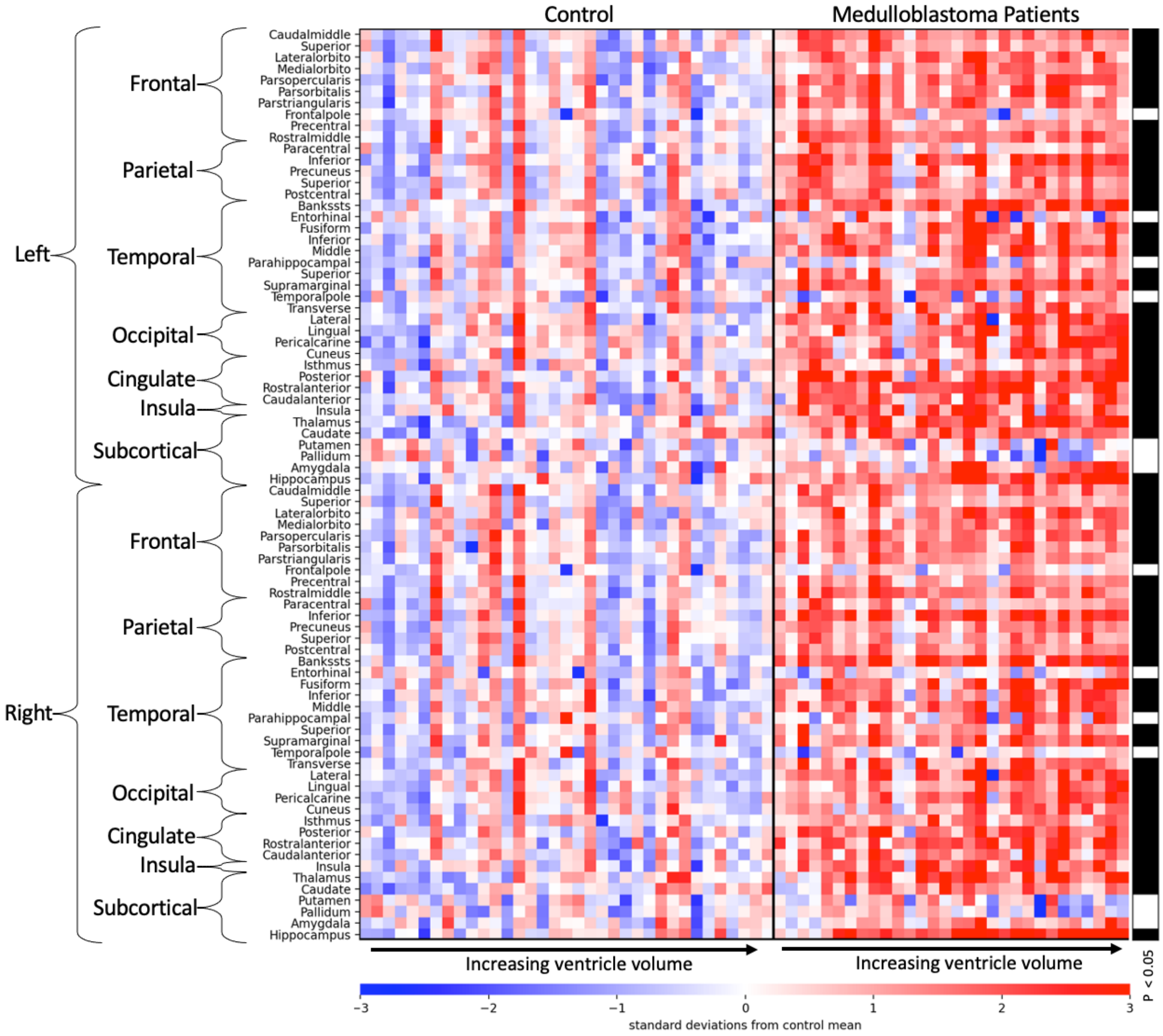

Supplementary Figure 4. Heatmap showing the deviation of radial diffusivity (RD) in each gray matter region of each healthy control and MB patient compared to the mean RD of the control group. In each group, subjects are aligned from left to right from smallest ventricular size to the largest ventricular size. The intensity of blue and red coloring indicates the standard deviation from the mean of the control group. The black color along the right side indicates that the difference from the mean of the control group is statistically significant ($p<0.05$) using a GLM that controls for age and ventricular volume.

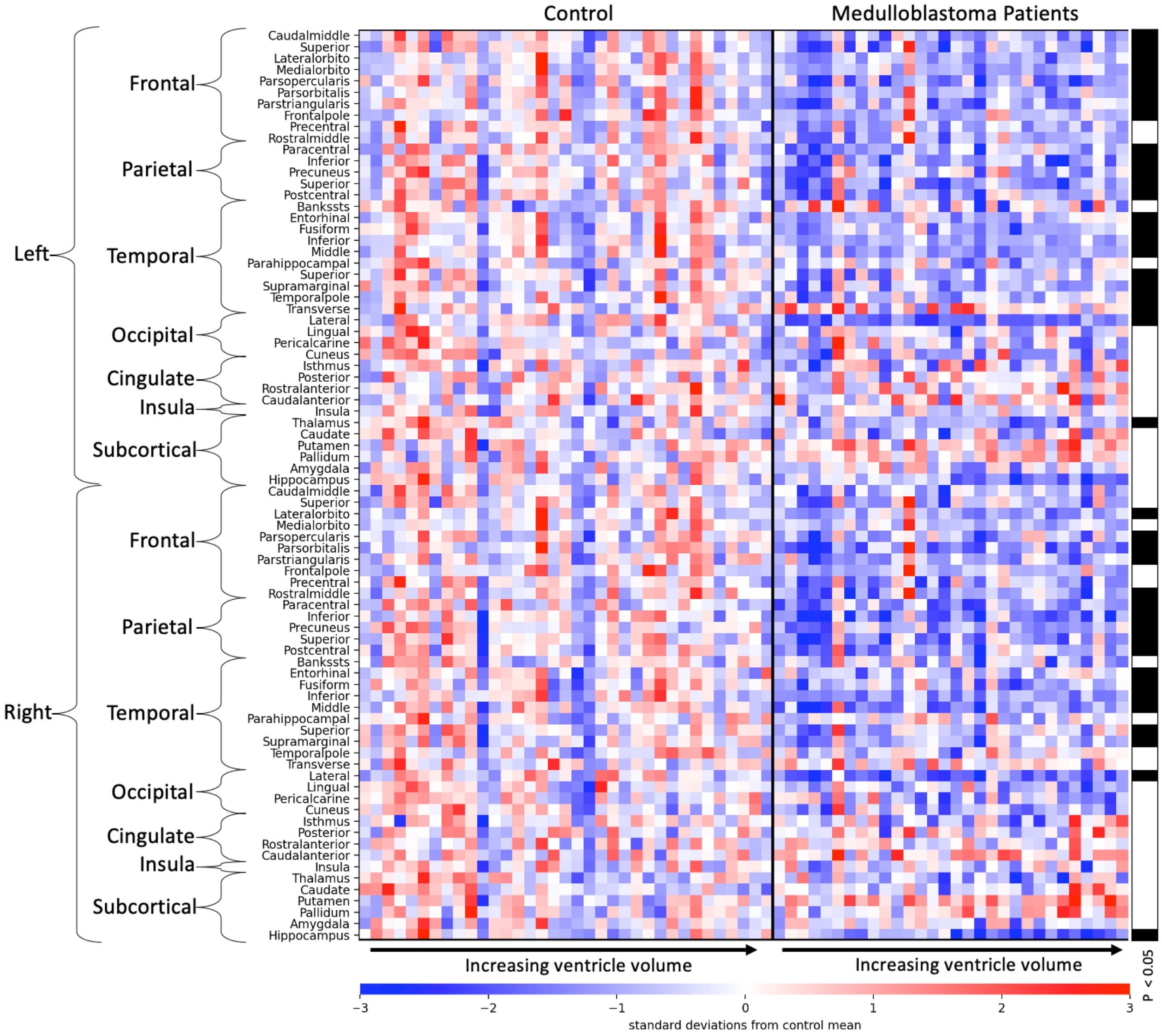

Supplementary Figure 5. Heatmap showing the deviation of fractional anisotropy (FA) in each gray matter region of each healthy control and MB patient compared to the mean FA of the control group. In each group, subjects are aligned from left to right from smallest ventricular size to the largest ventricular size. The intensity of blue and red coloring indicates the standard deviation from the mean of the control group. The black color along the right side indicates that the difference from the mean of the control group is statistically significant ($p<0.05$) using a GLM that controls for age and ventricular volume.

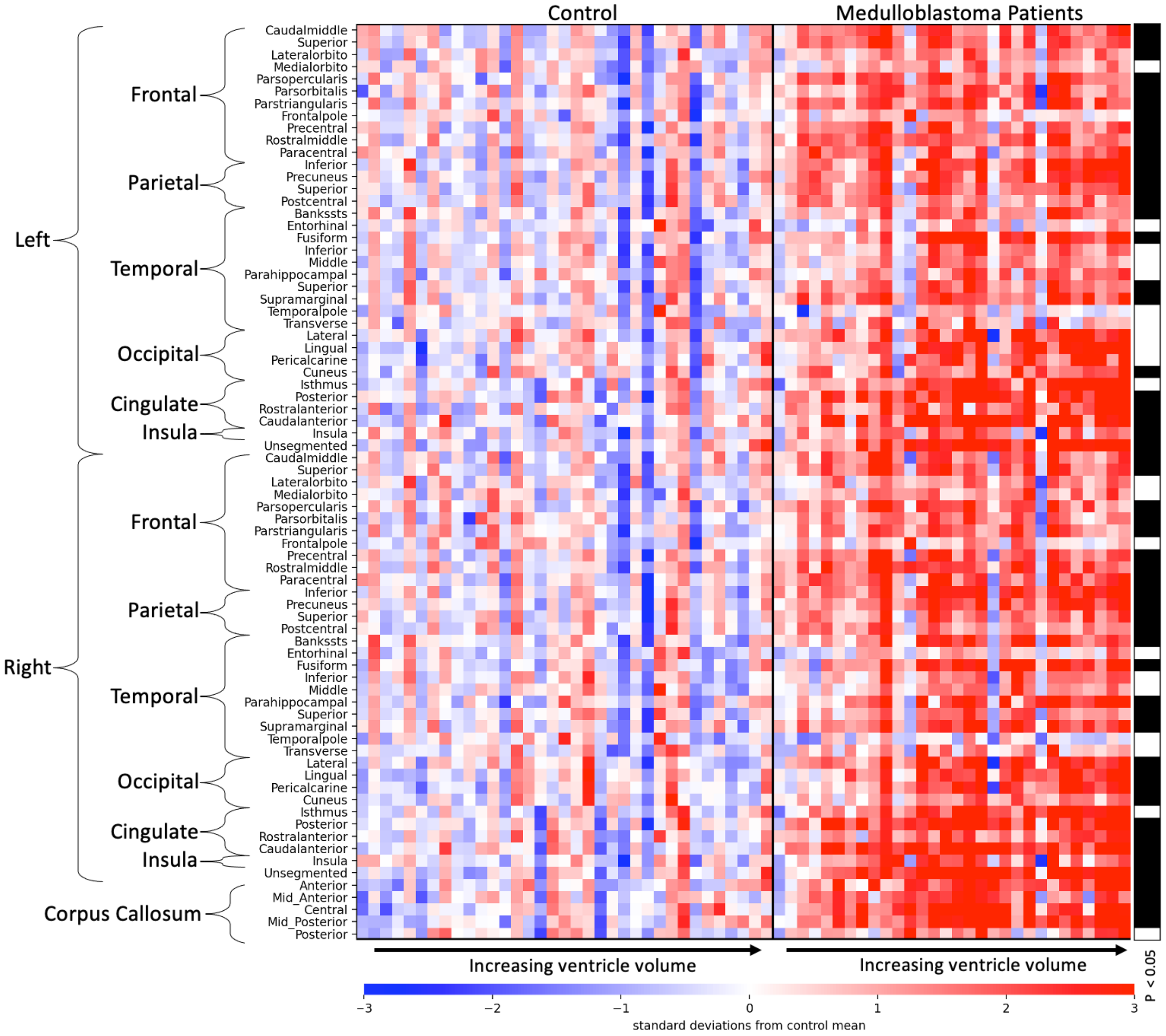

Supplementary Figure 6. Heatmap showing the deviation of mean diffusivity (MD) in each white matter region of each healthy control and MB patient compared to the mean MD of the control group. In each group, subjects are aligned from left to right from smallest ventricular size to the largest ventricular size. The intensity of blue and red coloring indicates the standard deviation from the mean of the control group. The black color along the right side indicates that the difference from the mean of the control group is statistically significant ($p<0.05$) using a GLM that controls for age and ventricular volume.

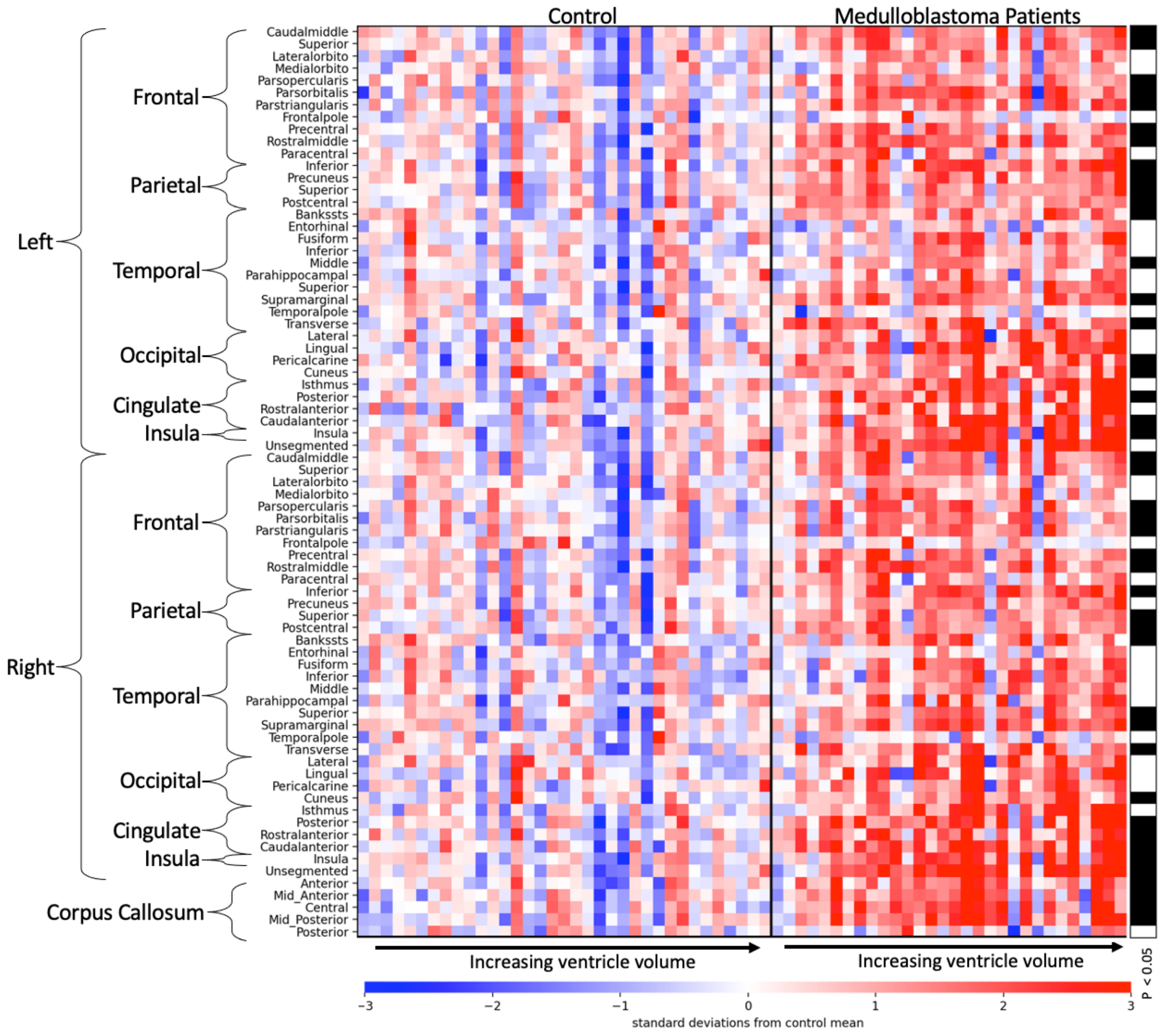

Supplementary Figure 7. Heatmap showing the deviation of axial diffusivity (AD) in each white matter region of each healthy control and MB patient compared to the mean AD of the control group. In each group, subjects are aligned from left to right from smallest ventricular size to the largest ventricular size. The intensity of blue and red coloring indicates the standard deviation from the mean of the control group. The black color along the right side indicates that the difference from the mean of the control group is statistically significant (p<0.05) using a GLM that controls for age and ventricular volume.

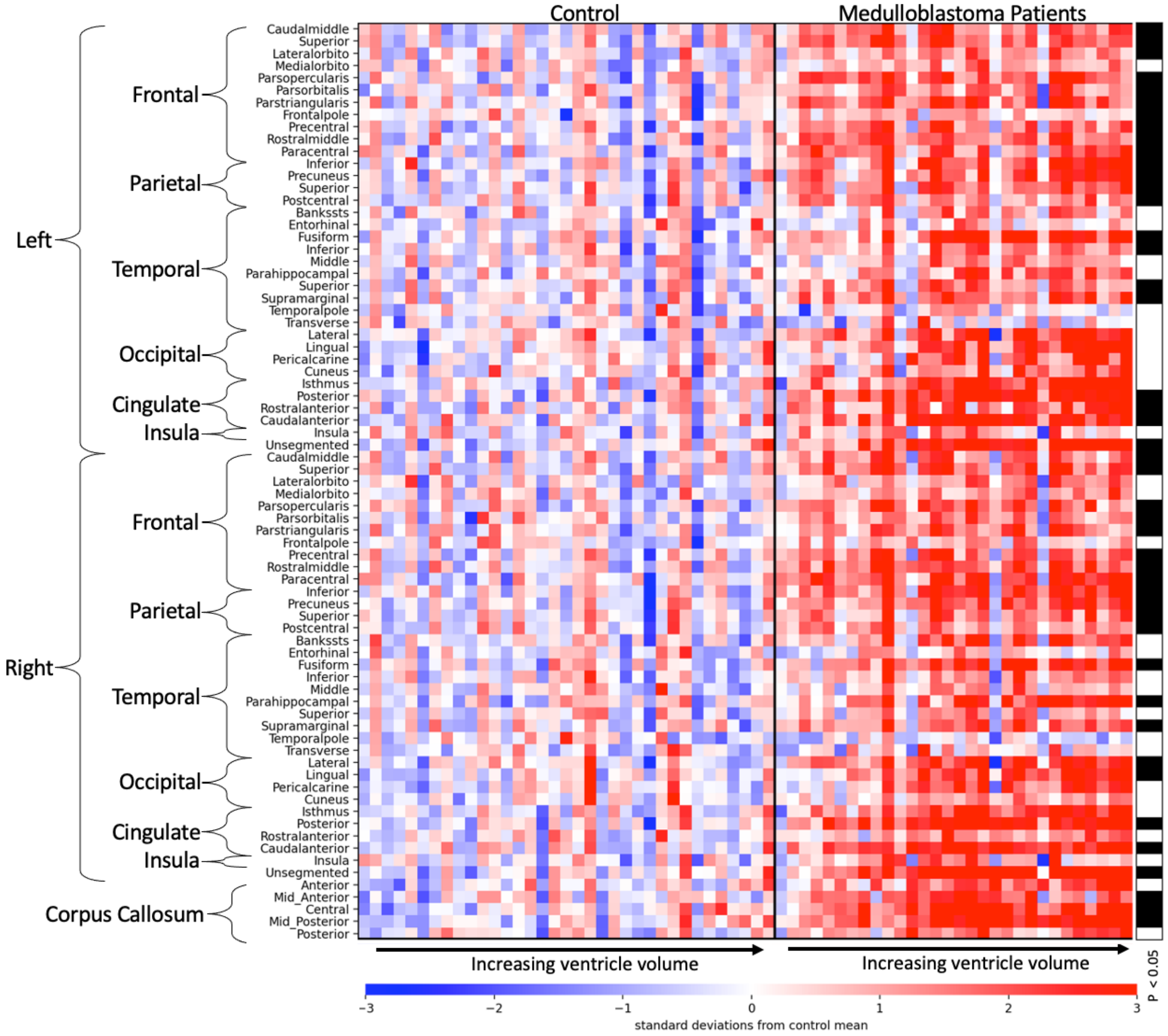

Supplementary Figure 8. Heatmap showing the deviation of radial diffusivity (RD) in each white matter region of each healthy control and MB patient compared to the mean RD of the control group. In each group, subjects are aligned from left to right from smallest ventricular size to the largest ventricular size. The intensity of blue and red coloring indicates the standard deviation from the mean of the control group. The black color along the right side indicates that the difference from the mean of the control group is statistically significant ($p<0.05$) using a GLM that controls for age and ventricular volume.

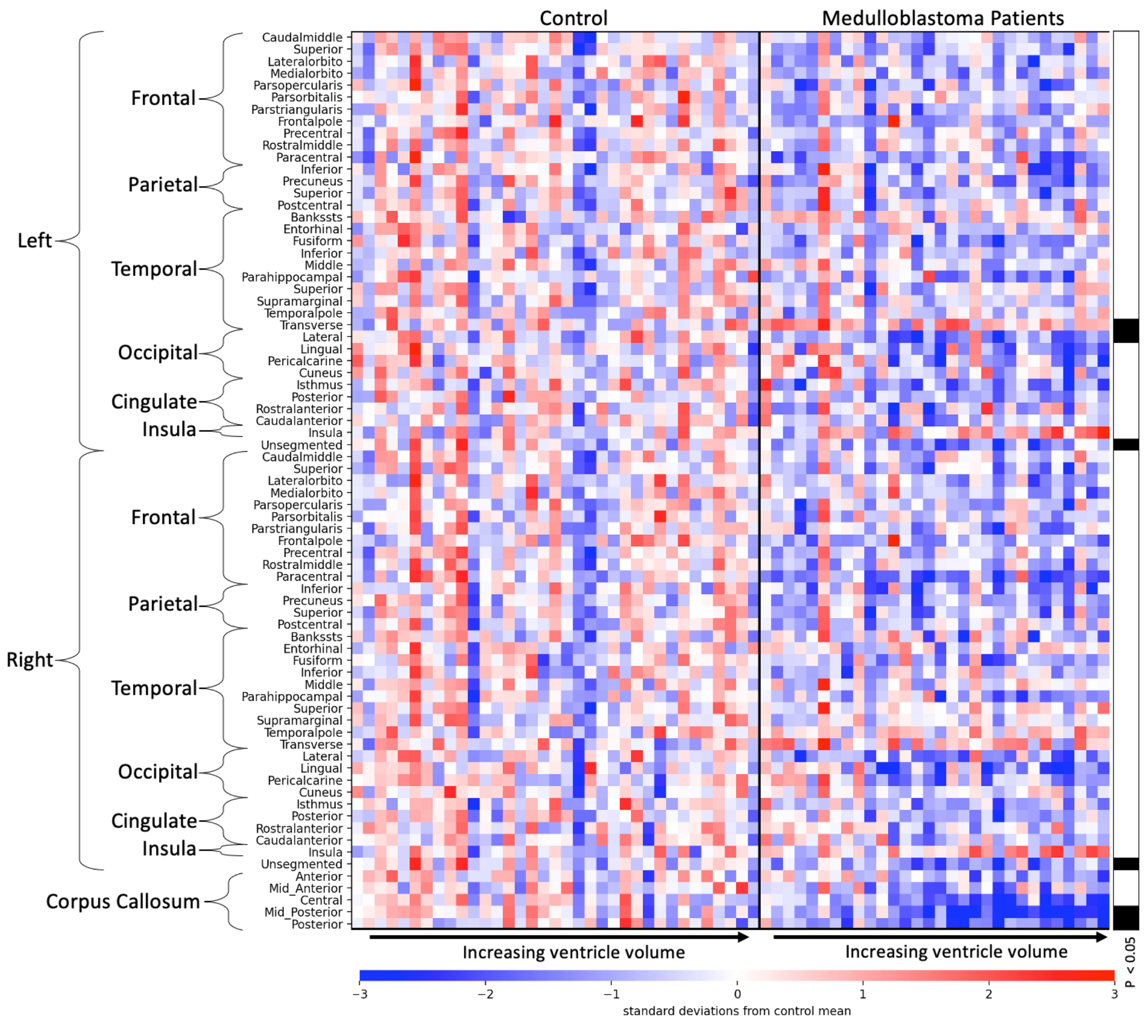

Supplementary Figure 9. Heatmap showing the deviation of fractional anisotropy (FA) in each white matter region in each healthy control and MB patient compared to the mean FA of the control group. In each group, subjects are aligned from left to right from smallest ventricular size to the largest ventricular size. The intensity of blue and red coloring indicates the standard deviation from the mean of the control group. The black color along the right side indicates that the difference from the mean of the control group is statistically significant (p<0.05) using a GLM that controls for age and ventricular volume.

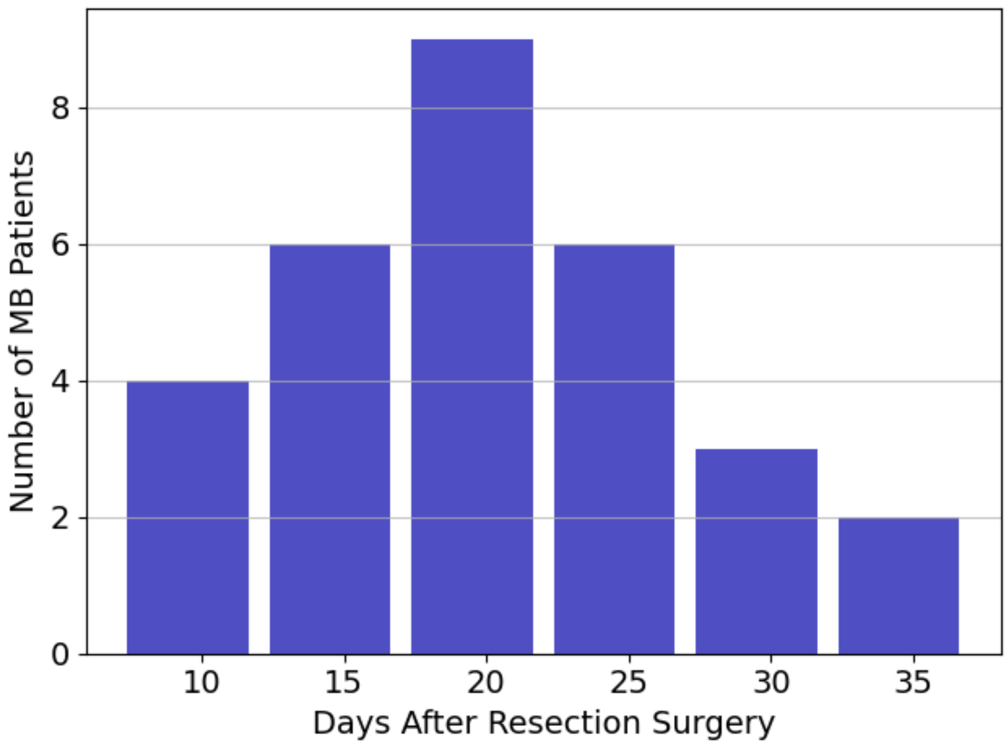

Supplementary Figure 10. Histogram of the interval between the date of surgery and the date of MRI acquisition.

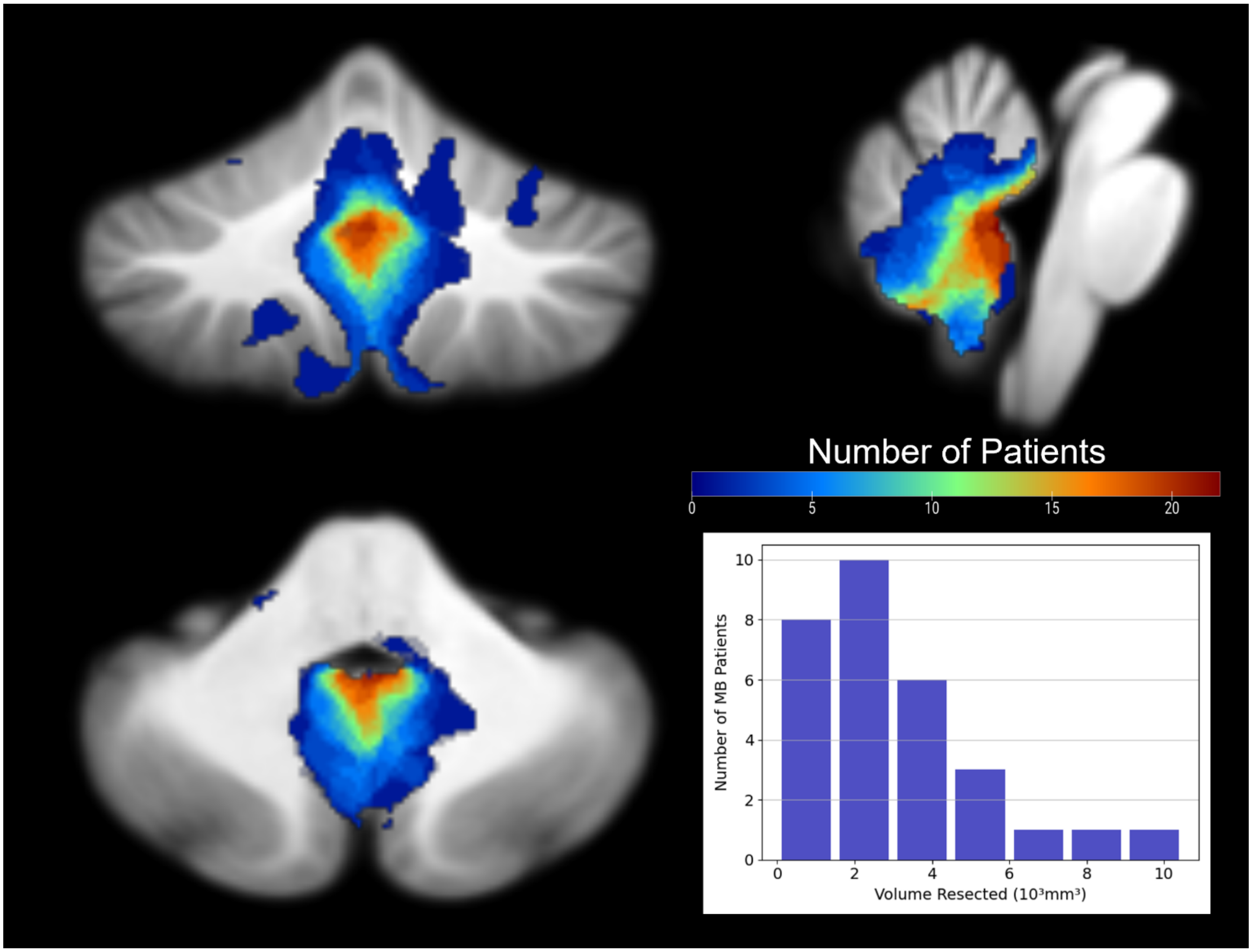

Supplementary Figure 11. Heat map showing the areas of the cerebellum resected in the medulloblastoma patient group. The midline superior vermis is resected in 24 of the 30 participants. Graph depicts the distribution of cerebellar volume resected.

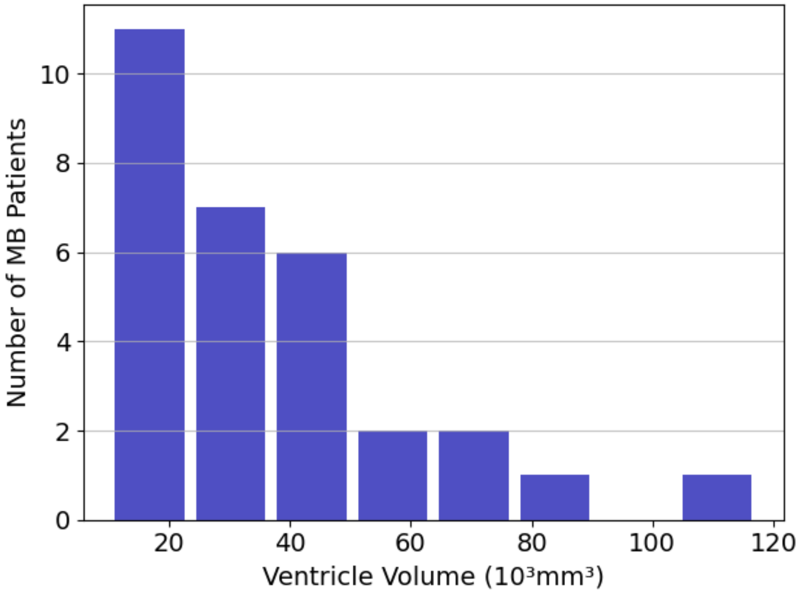

Supplementary Figure 12. Histogram showing the distribution of ventricular volume in the medulloblastoma group.

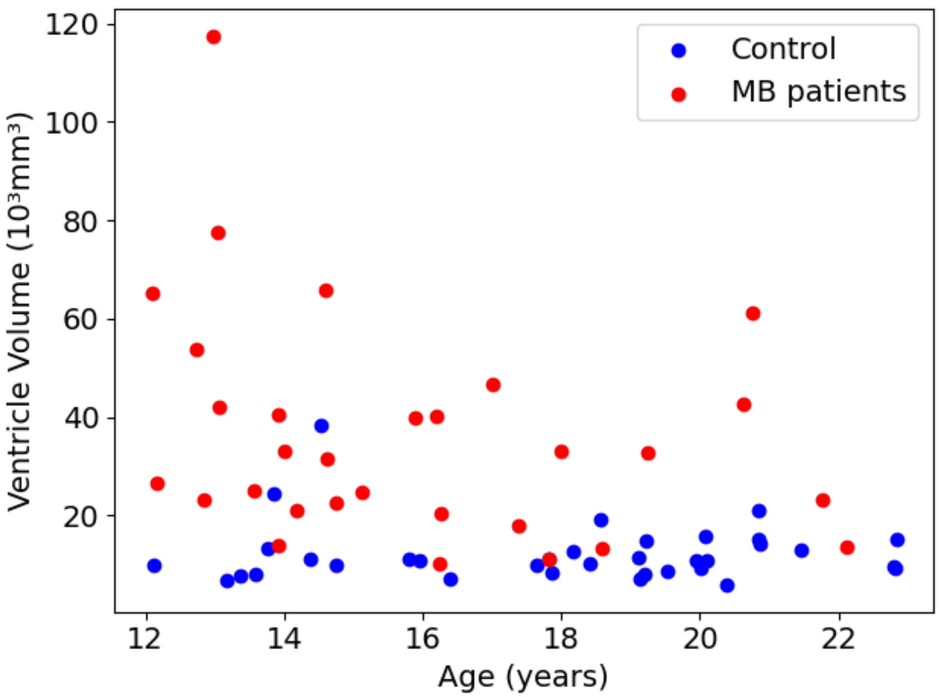

Supplementary Figure 13. Scatter plot of ventricular volume as a function of age in healthy control participants and patients with medulloblastoma. The ventricular volume is significantly larger in the medulloblastoma group ($p<0.05$) as shown through a t-test.

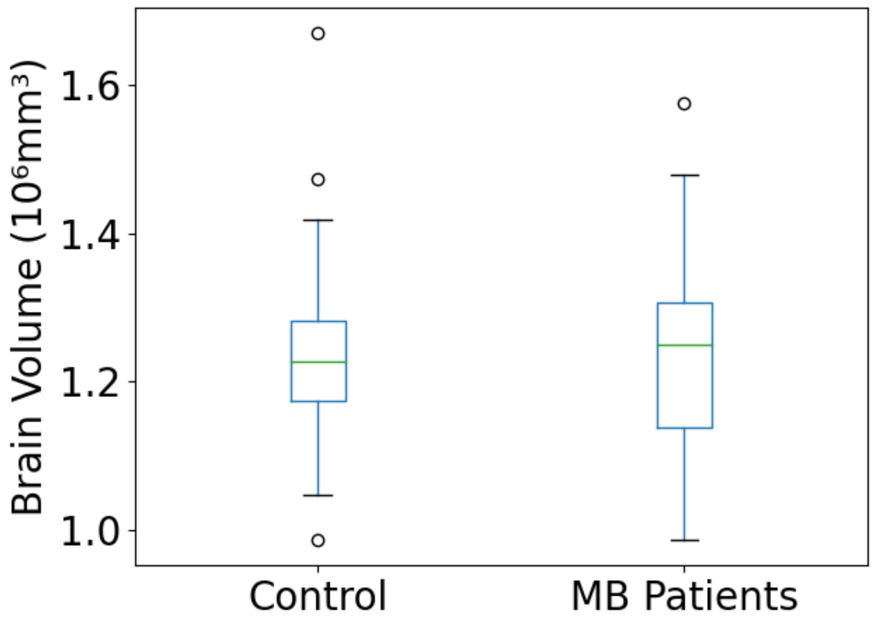

Supplementary Figure 14. Comparison of brain volumes between healthy control subjects and the medulloblastoma patients. Even though the age difference and ventricular volume between the groups were statistically significant, the brain volume was not different between the two groups as demonstrated by a t-test ($p>0.05$).

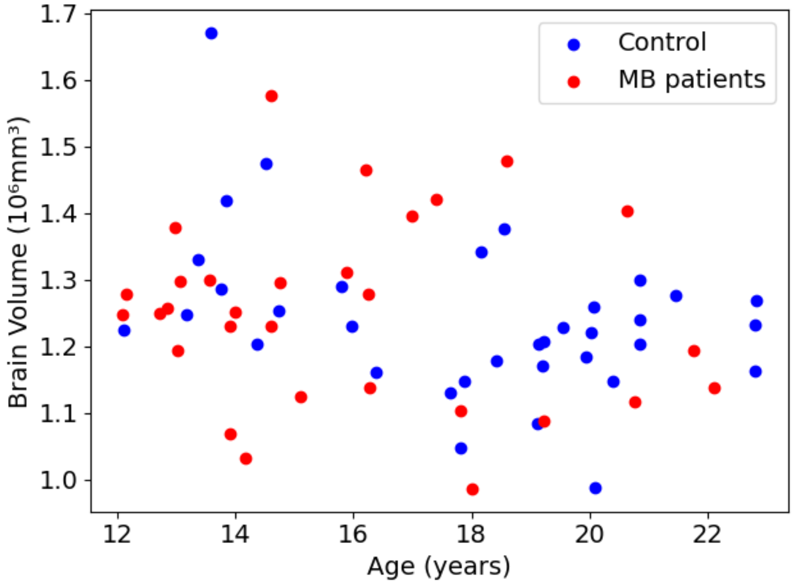

Supplementary Figure 15. Scatter plot showing the brain volume (excluding ventricles) as a function of age for both healthy control subjects and medulloblastoma patients. A t-test did not show significant differences between the groups ($p>0.05$).

Supplementary Table 1. Demography and tumor characteristics of patients with medulloblastoma

	**Age (in years)**	**Sex**	**Supratentorial Mets**	**Mets Size (in cm³)**	**Tumor subtype**	**Histology**	**Tumor volume (in cm³)**	**Presence of residual tumor**	**Ventricular volume (in cm³)**	**Presence of Periventricular edema**	**Treatment of hydrocephalus**	**Duration of symptoms at presentation (mo)**	**Location of the primary tumor**
1	17	F	No Mets		WNT	C	32.7	N§	33.04	N^	Y*	1	4th ventricle
2	16	M	No Mets		WNT	C	19.3	N	46.52	N	Y	2	4th ventricle
3	19	F	No Mets		WNT	C	24.1	N	32.78	N	N!	3	Vermis
4	16	F	No Mets		WNT	C	21.2	N	11.03	N	N	16	Vermis
5	17	M	Infundibular recess Mets	0.8	WNT	C	40.3	N	17.97	N	N	18	MCP
6	12	F	No Mets		WNT	C	24.4	N	26.47	N	Y	<1	4th ventricle
7	21	F	No Mets		SHH	LA	29.2	N	23.11	N	N	1	Cerebellar hemisphere
8	14	M	No Mets		SHH	LA	34.2	N	31.49	N	Y	2	Cerebellar hemisphere
9	22	M	Rt sylvian , Lt frontal lobe mets	<0.5	SHH	C	26.3	N	13.60	N	Y	3	Cerebellar hemisphere
10	18	M	Cingulate sulcus, L occipital Mets	0.8	SHH	DN	48	N	13.26	N	N	<1	Cerebellar hemisphere
11	20	M	No Mets		SHH	DN	28.1	N	42.49	N	N	<1	Vermis
12	13	M	No Mets		NWNS	C	46.4	N	33.02	N	N	1	Vermis
13	14	F	No Mets		NWNS	C	31.8	N	20.89	N	Y	1	MCP
14	15	M	No Mets		NWNS	C	32.9	N	39.82	N	Y	1	4th ventricle
15	16	M	Infundibular recess Mets	<0.5	NWNS	C	24.1	N	20.37	N	Y	2	4th ventricle
16	12	M	Infundibular recess Mets	1.6	NWNS	C	45.7	N	65.19	N	Y	2	4th ventricle

17	12	F	No Mets		NWNS	C	26.1	N	77.42	N	Y	3	4th ventricle
18	15	F	Lt sylvian fissure Mets	0.6	NWNS	C	10.5	N	24.60	N	N	3	4th ventricle
19	13	M	No Mets		NWNS	LA	31.8	N	40.57	N	Y	3	4th ventricle
20	14	M	Infundibular recess Mets	0.5	NWNS	LA	36.7	N	22.67	N	Y	4	4th ventricle
21	14	F	No Mets		NWNS	C	40.3	N	65.87	N	N	5	4th ventricle
22	12	M	No Mets		NWNS	C	39.3	N	53.85	N	Y	6	4th ventricle
23	12	M	Infundibular recess NMets	1.1	NWNS	C	31.7	N	117.39	N	Y	12	4th ventricle
24	12	M	No Mets		NWNS	C	5.3	N	23.05	N	N	<1	4th ventricle
25	13	M	No Mets		NWNS	C	21.8	N	14.03	N	N	<1	Inferior vermis
26	13	M	No Mets		NWNS	LA	37.1	N	25.09	N	N	<1	4th ventricle
27	16	M	No Mets		NWNS	C	10.2	N	40.06	N	N	<1	4th ventricle
28	12	F	No Mets		NWNS	C	28.7	N	42.15	N	N	1	4th ventricle
29	20	F	No Mets		I	Myo	43.6	N	61.26	N	N	2	4th ventricle
30	16	M	No Mets		I	Mln	22.4	$Y^{§}$	10.06	N	N	2	Vermis

C, Classic Histology; DN, desmoplastic/nodular I-Indeterminate; LA, Large cell/anaplastic; mln, Melanotic differentiation; Myo, Myogenic differentiation; $N^{§}$, absent residual tumor; N^, Absent of periventricular edema; $N^{!}$, No treatment of hydrocephalus; NWNS, Non-WNT non-SHH; SHH, *SHH*-activated; WNT,*WNT*-activated; $Y^{§}$, residual tumor present, volume $2.9cm^3$; Y*, hydrocephalus was treated with ventriculostomy but the catheters were removed at the time of imaging.

Supplementary Table 2. Comparison of the IQ Value between the Control and MB patients

	Group			
	Control (N=35)	MB Patients (N=21)	Total (N=56)	P-value
**IQ**				0.3471[1]
N	35	21	56	
Mean (SD)	103.7 (11.20)	97.9 (22.36)	101.5 (16.35)	
Median	103.0	94.0	103.0	
Range	76.0, 128.0	58.0, 144.0	58.0, 144.0	

[1]Wilcoxon rank sum p-value;

Supplementary Table 3. Handedness of the control subjects and MB patients

**CONTROL SUBJECTS**	**Dominant Hand**	**MB PATIENTS**	**Dominant Hand**
1	R	1	R
2	R	2	R
3	R	3	R
4	R	4	R
5	R	5	R
6	R	6	R
7	R	7	U
8	L	8	R
9	R	9	R
10	R	10	R
11	R	11	R
12	R	12	R
13	R	13	R
14	R	14	R
15	R	15	U
16	R	16	R
17	R	17	R
18	R	18	R
19	R	19	R
20	R	20	R
21	R	21	R
22	R	22	R
23	R	23	R

24	R	24	R
25	R	25	R
26	R	26	R
27	R	27	R
28	R	28	R
29	R	29	R
30	R	30	A
31	R		
32	R		
33	R		
34	R		
35	R		

A: ambidextrous, patient became right handed after diagnosis and was left handed before diagnosis; L, left hand; R, right hand; U unknown.

Supplementary Table 4. Distribution of the magnets for MRI acquisition.

	Control	MB Patients
Skyra	21	5
Prisma	14	25

The FA and MD of the entire gray matter and entire white matter were compared between the control subjects scanned in the Skyra system (n=21) with those scanned on the Prisma system (n=14), after controlling for the age. There were no statistically significant differences between the scanners in either of FA or MD (p>0.05).

Supplementary Table 5. Significant association between time interval (between surgery and MRI acquisition) and specific microstructural alterations in specific brain regions. There is no significant association between time interval and any DTI parameters in rest of the brain areas. (FA, fractional anisotropy; MD, mean diffusivity, RD, radial diffusivity)

DTI parameter	**Brain regions**	**p-value**
MD	Right transverse temporal white matter	.02295
FA	Right rostral middle frontal gray matter	.04936
FA	Right transverse temporal gray matter	.01416
FA	Right transverse temporal white matter	.02493
RD	Left transverse temporal gray matter	.01654
RD	Left transverse temporal white matter	.04611
RD	Right transverse temporal white matter	.00265
